# CD16 Expression on Monocytes in Healthy Individuals but Not Schistosome-Infected Patients Is Positively Associated with Levels of Parasite-Specific IgG and IgG1

**DOI:** 10.1371/journal.pntd.0003049

**Published:** 2014-08-07

**Authors:** Laura J. Appleby, Norman Nausch, Louise Erskine, Claire D. Bourke, Nadine Rujeni, Nicholas Midzi, Takafira Mduluza, Francisca Mutapi

**Affiliations:** 1 Institute of Immunology & Infection Research, Centre for Immunity, Infection & Evolution, School of Biological Sciences, University of Edinburgh, United Kingdom; 2 National Institutes of Health Research, Harare, Zimbabwe; 3 Department of Biochemistry, University of Zimbabwe, Harare, Zimbabwe; René Rachou Research Center, Fiocruz, Belo Horizonte, Brazil, Brazil

## Abstract

Human IgG1 antibody responses are associated with protection against *Schistosoma haematobium* infection and are now a target for schistosome vaccine development. This study aimed to investigate the relationship between total IgG and the IgG subclasses and the monocyte IgG receptor, known as FcγRIIIa or CD16, in schistosome exposed people. Systemic levels of schistosome-specific anti-adult worm total IgG and IgG subclass titres were measured by ELISA in 100 individuals from an *S. haematobium* endemic area in Zimbabwe and, using parametric statistical methods and regression analysis, related to the levels of CD16 expression on individuals' circulating monocytes, determined via flow cytometry. Monocyte CD16 expression rose with parasite-specific total IgG and IgG1 in healthy participants, but not in schistosome infected patients. Similar to parasite-specific IgG and IgG1, CD16 expression in healthy individuals is associated with protection against schistosome infection. This relationship indicates a mechanistic link between the innate and adaptive immune responses to helminth infection in protection against infection. Further understanding the elements of a protective immune response in schistosomiasis may aid in efforts to develop a protective vaccine against this disease.

## Author Summary

Schistosomiasis is a parasitic disease caused by the parasite *Schistosoma* spp. Over 240 million people are infected worldwide, mainly in Sub-Saharan Africa, but an efficacious, protective vaccine has yet to be found. Protection against schistosome infection in individuals living in endemic areas is mediated by antibodies. In particular, IgG1 antibody has been shown to be protective against infection in individuals living in endemic areas, and eliciting IgG1 production has become a cornerstone of vaccine development efforts. However, little is known about the mechanisms by which IgG1 induces protection. The cell surface molecule CD16 is an IgG antibody receptor expressed on monocytes and binds preferentially to IgG antibody subclasses. The work presented here thus investigates the relationship between IgG levels and the monocyte CD16 receptor in a population endemically exposed to infection with schistosomes. We present results linking CD16 expression with IgG1 levels, whereby uninfected individuals have a positive relationship between IgG1 and CD16 expression levels, while schistosome infected individuals did not show any statistically significant relationship between the two. Thus we provide evidence to suggest a mechanistic link between the innate and adaptive immune response in parasitic infection, associating monocyte CD16 expression with a protective immune response.

## Introduction

An estimated 200 million people worldwide are infected with helminths of the genus S*chistosoma*, with the heaviest burden of disease occurring in sub-Saharan Africa, where both *Schistosoma haematobium* and *Schistosoma mansoni* are endemic, causing significant morbidity amongst affected communities [1]. Infection and disease are controlled by treatment with the drug praziquantel (PZQ), and the World Health Organization (WHO) recommends protective chemotherapy via mass drug administration (MDA) with PZQ in endemic areas [2]. There is mounting pressure to develop a vaccine against schistosomiasis, which would provide long term protection to the 650 million people at risk of exposure [3], and pre-empt the development of drug resistance. Current vaccine development research focuses on determining which naturally developed immune responses are associated with protective immunity that develops in the context of endemic exposure to infection, and investigate ways of inducing those responses artificially whilst avoiding a pathological response [4], [5]. While significant progress has been made in characterising humoral and cellular responses in experimental models, relatively less work has been conducted relating the innate and adaptive arms of the immune system in schistosome infected versus uninfected humans. In particular, there is a paucity of studies simultaneously determining cellular and related humoral responses associated with natural protection against schistosome infection.

Experimental studies have shown links between innate cells from the myeloid lineage and resistance to helminth infection. For example, murine macrophages and are involved with tissue repair and fibrosis [6], [7], as well as in limiting pathology by regulating Type 2 cytokine production [8], [9] and inhibiting T cell proliferation [10]. This current study focused on circulating monocytes, myeloid cells related developmentally to macrophages, which are present in the blood vessels and are thus easily accessible for investigation in humans. Studies from several decades ago showed a direct role ex vivo for human PBMC-derived monocytes in the killing of schistosomula [11]–[13]. Similar to macrophages, monocytes display phagocytic capabilities and express varying levels of the FcγRIIIa (also known as the CD16 receptor) [14], which is related to distinctions in their phenotype and function in a range of pro-inflammatory conditions [15], [16]. The Fcγ receptors have a critical role in immune regulation, acting as a link between the humoral and innate cellular arms of the immune response [17]. In humans, the CD16 receptor exhibits high affinity binding to the Fc portion of IgG antibodies, with high affinity binding demonstrated to IgG1 and IgG3, which leads to phagocytosis, release of inflammatory mediators and clearance of immune complexes [14]. The importance of the interaction between IgG and Fcγ receptors has been demonstrated in experimental models, whereby there is a diminished macrophage effector function induced after IgG1-mediated phagocytosis in Fcγ chain knock-out mice [18]. Furthermore, *S. mansoni* infection exacerbated granuloma formation and fibrosis in both Fcγ receptor and in B cell deficient mice [19], highlighting the importance of antibody signalling via the Fcγ receptor in protection against pathology associated with schistosomiasis infection. However, there are few studies relating IgG subclasses to the Fcγ receptors in human schistosomiasis. To address this knowledge gap, the present study focuses on the relationship between CD16 and the IgG subclasses.

Our previous studies, and those of others, have shown that, in humans, schistosome-specific IgG1 and IgG3 antibodies are associated with natural resistance to infection [20]–[22]. Induction of helminth-specific IgG1 and IgG3 through vaccination is now preferred over IgE to avoid generating IgE-mediated pathological responses to vaccination [4], [23] and, in particular, this study focuses on these protective subclasses.

This study, therefore, investigated the relationship between levels of the IgG receptor, FcγRIIIa (CD16) and schistosome specific IgG subclasses in uninfected, healthy individuals versus schistosome infected patients. The healthy individuals comprised of young people, who had yet to acquire schistosome infection, and older people who were putatively resistant to infection, as they were infection free despite being lifelong residents of the schistosome endemic areas and experiencing regular exposure to infective water. The study focused on adult schistosome-specific IgG responses since adult worms reside in the circulating blood, and thus are in direct contact with monocytes in this compartment. In addition, our studies, as well as those of others, have highlighted the importance of the adult worm stage in stimulating protective immune responses [24]–[27].

## Methods

### Ethical approval

Ethical and institutional approval was granted by the Medical Research Council of Zimbabwe and the University of Zimbabwe's Institutional Review Board. Local permission for the study was granted by the Provincial Medical Director. The study design, aims and procedures were explained in the local language, Shona, prior to enrolment. Participants were free to drop out of the study at any time and informed written consent/assent was obtained from all participants and/or their guardians prior to taking part in the study and to receiving antihelminthic treatment.

### Study design

The study presented here was part of a larger on-going immuno-epidemiological study based in Mashonaland East, Zimbabwe where *S. haematobium* is endemic [28]. The area has a low prevalence of soil transmitted helminths (STH) and *Schistosoma mansoni* [29], and the residents are subsistence farmers with frequent contact with infected water for purposes of bathing, washing and collecting water. Recruitment into the study was school based and the wider community was also invited to participate. Residential history, antihelminthic treatment history and water contact habits of the participants were captured through questionnaire. Following sample collection, participants were offered treatment with the antihelminthic drug praziquantel at the recommended dose of 40 mg/kg of body weight [3].

### Inclusion criteria

In order to be included in this study participants had to meet the following criteria: 1) be lifelong residents of the study area to allow age to be used as a proxy for history of exposure to schistosome infection, 2) have provided a minimum of two urine and two stool samples on consecutive days for parasite detection, 3) not have previously received antihelminthic treatment, 4) be negative for co-infection with malaria, STH, *S. mansoni* and HIV and 5) have provided a blood sample for serological and cellular assays. Further to this, participant's PBMC sample must have yielded at least 10^6^ cells to allow enough cells for all experimental conditions. From an initial cohort of 633 recruited individuals, 68 were excluded for not meeting criteria 1–4 above and a further 184 did not provide sufficient blood sample for both serological assays and cell phenotyping. From the remaining 381 individuals, a cohort of 100 individuals was further selected to allow for, as far as possible, equal numbers of females to males and an even distribution of ages and infection prevalence. Individuals with one or more *S. haematobium* eggs found in their urine samples were classified as infected. The final study group was divided into three age groups and is described in Table 1.

**Table 1:** Characteristics of study cohort.

	*Age Group*					
	5–10 years		11–15 years		>16 years	
*Infection Status*	Sh−	Sh+	Sh−	Sh+	Sh−	Sh+
Sample size (no.)	28	16	18	13	16	9
Mean Age (years)	7.68	7.62	13.17	12.54	30.12	27.11
Infection intensity	0	56.78	0	99.44	0	59.26
Infection range (SD)	0	1.33–297 (83.7)	0	0.33–523 (165.7)	0	0.33–550 (109.5)
Males∶Females	8∶20	11∶5	9∶9	8∶5	2∶14	3∶6

All selected people of the cohort were negative for HIV, soil transmitted helminths and *S. mansoni*; infection intensity: eggs/10 mL urine; Sh− negative for *S. haematobium*, Sh+ positive for *S. haematobium*.

### Sample collection

From each participant a stool and urine sample was collected on three consecutive days and examined microscopically for the presence of *S. haematobium* eggs in urine, and *S. mansoni* and STH eggs in stool using standard techniques [30], [31]. A random sample of 100 stool samples was also processed via the formol ether concentration technique, and these confirmed Kato Katz diagnosis [32]. Up to 20 millilitres of venous blood was collected from each participant in heparinised tubes or silicone –coated tubes (both from BD Biosciences, San Jose, CA), for purposes of processing for PBMC purification (heparin tubes), or serum (silicone tubes) using routine methods. An additional drop of blood was collected from each participant for microscopic detection of malaria parasites and for HIV detection using DoubleCheckGold HIV 1&2 Whole Blood Test (Orgenics Ltd., Yavne, Israel). Peripheral blood mononuclear cells (PBMC) were isolated from the remaining tubes via density gradient centrifugation using Lymphoprep (Axis-Shield, Cambridgeshire, UK). Isolated PBMCs were cryopreserved and stored in liquid nitrogen in Zimbabwe prior to freighting to Edinburgh in dry shippers where the overall viability of isolated PBMCs was estimated in a sample of 84 individuals' PBMCs using propidium iodide (PI) (Sigma-Aldrich, Dorset, UK) exclusion. Mean PI uptake in this sample was 15.3% (+/−1.06%), which is within the range considered viable.

### Antibody assays

Schistosome soluble worm antigen preparation (SWAP)-specific antibody serum levels for total IgG, IgG1, IgG2, IgG3 and IgG4 were quantified using antibody ELISA. Lyophilized SWAP (Theodor Bilharz Institute, Giza, Egypt) was reconstituted as recommended by the manufacturer and as described by Mutapi *et al.* [25]. ELISAs were conducted as previously reported [22], using 5 µg/ml of SWAP antigen in carbonate bicarbonate buffer to coat all ELISA plates, and adding sample at a 1∶100 dilution in 5% skimmed milk. Secondary IgG HRP-conjugated antibody was added at a 1∶1000 dilution for total IgG and IgG1, and at a 1∶500 dilution for IgG2, IgG3 and IgG4. The colorimetric reaction was left for 10 minutes for total IgG, and 15 minutes for the IgG subclasses, and quantified with an ELISA reader at 405 nm. Each antibody ELISA was performed in duplicate on the same day for all samples with positive and negative controls on each plate.

### Phenotyping of monocytes

Cryopreserved PBMCs were thawed as previously described [33], and resuspended at 5×10^6^ cells/ml in PBS. Cells were incubated with 10% FCS at 4°C for 10 minutes prior to staining for 30 minutes with Alexa488 conjugated anti-CD14 (clone M5E2), PE-Cy7 conjugated HLA-DR (clone L243) (both from BD Biosciences, San Jose, CA), and Pacific Blue conjugated anti-CD16 (clone CB16) (eBiosciences, San Diego, CA). Unbound antibodies were washed off and cells were resuspended in PBS prior to acquisition of at least 50,000 live events on a BD FACS LSR II (BD Biosciences, San Jose, CA). Compensation was performed prior to acquisition of each experiment using BD FacsComp beads (BD Biosciences, San Jose, CA). Analysis was performed using FlowJo software (TreeStar, USA) and mean fluorescence intensity (MFI) was calculated for CD16.

To ensure that only CD14 positive cells representing monocytes were analysed, only cells expressing both HLA-DR and CD14 were selected for analysis, in a gating strategy previously described [28], [34]. Briefly, a live gate to include all leukocytes was drawn based on forward scatter (FSC) and side scatter (SSC), HLA-DR positive cells were gated to exclude any CD16+ expressing natural killer (NK) cells as well as other non-MHC expressing cells. Monocytes were defined as CD14 or CD16 expressing cells. The expression level of CD14 and CD16 was reported as MFI.

### Statistical analysis

All statistical analyses were conducted using the statistical package SPSS version 19 (IBM Corp, NY, USA). Due to the possibility of gender and age dependent exposure patterns in this population [35], [36], appropriate statistical techniques were necessary to adjust for this variation prior to investigating the relationship of interest [37]. Parametric statistical modelling in the form of analysis of variance (ANOVA) and linear regression was therefore used. Data were transformed in order to meet assumptions of parametric tests. Surface marker expression (measured as MFI) was log transformed (log_10_(x+1)). Antibody level (after subtraction of the blank control) was square root transformed. Categorical variables were sex (male/female), infection status (uninfected/infected) and age group (5–10 years [age group where infection is rising], 11–15 years [age group where infection is peaking] or >16 years [age group where infection is declining]).

To determine the extent of changes in the proportion of monocytes relative to the rest of the PBMCs, ANOVA with sequential sums of squares (SS) was used with the total number of live monocytes as the dependent variable, and the independent variables were sex (male or female), age group (5–10 years, 11–15 years or >16 years) and infection status (uninfected or infected).

Monocytes from different individuals show varying levels of CD14 and CD16 expression intensity dependent on various factors including age [38], [39] and presence of inflammation [40]. Therefore, to test the hypothesis that CD14 and CD16 receptor expression levels changed with infection and age group, a multivariate analysis of variance (MANOVA) with sequential SS was used with receptor expression (CD14 and CD16) as the dependent variable, and the independent variables were sex, age group and infection status. The model was extended to include an interaction term between age and infection to assess patterns of surface receptor expression co-dependent on age and infection status. The relationship between CD16 expression and age group dependent on infection status was investigated further using a partial correlation, controlling for variation due to sex, and followed with Fisher's z transformation, which tests for a significance in the difference between two correlations.

The relationship between each of the anti-SWAP antibody titres (total IgG, IgG1, IgG2, IgG3, IgG4) with infection and age group was tested using a univariate ANOVA with sequential SS, entering sex and age group before infection in the analysis. In addition, the interaction between infection and age was tested and all appropriate post-hoc tests were conducted.

Finally to determine the relationship between CD16 expression on monocytes and antibody levels according to infection status, a linear regression analysis was used. The relationship between antibody levels and CD16 expression according to infection status was investigated after accounting for sex and age differences, and significant interactions were followed up with a correlation analysis of the relationship between the two variables in uninfected and infected individuals separately.

For all statistical tests significance of p≤0.05 was considered significant.

## Results

### The proportion of total circulating monocytes in schistosome patients is similar to that in healthy participants

**Figure 1:**
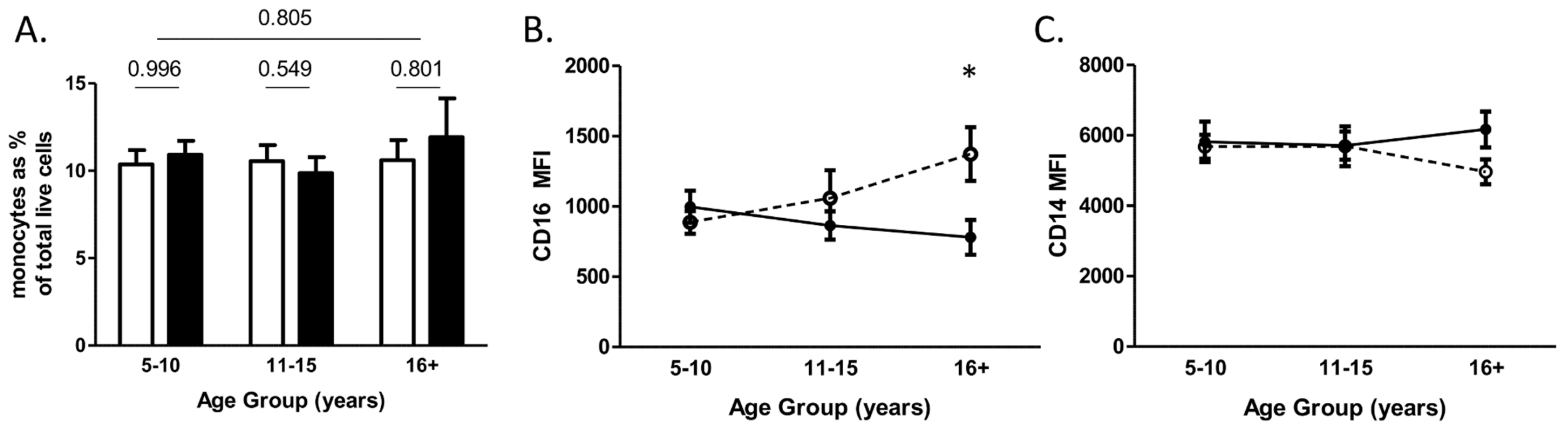
CD16 but not CD14 expression intensity on circulating monocytes differs between uninfected and infected older patients. Proportion of (A) total circulating monocytes, (B) MFI of CD16, and (C) MFI of CD14 on monocytes according to age group and infection status and as determined by flow cytometry. Open bars and open circles with dashed lines, healthy individuals; filled bars and closed circles with solid lines, schistosome infected patients. Bars represent standard error of mean. Significant differences between infected and uninfected participants within age groups according to sub-group analysis are indicated by *(p≤0.05).

The proportion of monocytes in PBMCs did not vary significantly with host age or infection as shown in Figure 1A. Thus, any changes observed in subsequent analyses will be a result of changes in monocyte phenotype between individuals, rather than as a result of changes in monocyte proportions, for example due to migration of monocytes into or out of the vasculature.

### Healthy older individuals express significantly higher monocyte surface CD16 than schistosome infected patients of the same age

The expression levels of CD14 and CD16 on monocytes were investigated with relation to infection status. CD16 expression levels were shown to vary dependent on age and infection status (Table 2 and Figure 1B). Thus, while expression of CD16 on monocytes was similar between infected (schistosome patients) and uninfected (healthy) individuals in the younger age groups, with increasing age there was an increasing intensity of expression in the healthy individuals compared to a decreasing intensity of expression with age in the infected individuals as shown in Figure 1B. Consistent with the heterogeneous relationship between age and infection status, the correlation coefficient was significantly different (z = 2.97, p = 0.003) with schistosome patients having a positive relationship (r = 0.350, p = 0.006) and healthy individuals having a negative relationship (r = −0.287, p = 0.085). The oldest age group demonstrated significant differences in monocyte CD16 expression dependent on infection status (Figure 1B). In contrast, levels of the monocyte marker CD14 did not vary with any of the investigated variables (Table 2 and Figure 1C).

**Table 2:** Relationship between the MFI of CD14 or CD16 expression on monocytes with infection status and age group.

	*Monocyte marker*	
*Independent variable*	*CD16*	*CD14*
Sex	**5.370 (M<F)**	0.235 (M>F)
Age group	0.813 (1<2<3)	0.157 (1>2<3)
Infection status	0.60 (sh−>sh+)	0.80 (sh−<sh+)
Infection status * Age group	**4.946**	0.772

F values of output of MANOVA for relationship between CD14 or CD16 and each factor. Model accounts for variation in sex (male (M) or female (F)), age group (1: 5–10 years; 2: 11–15 years; 3: >16 years), and infection status (uninfected: sh−; infected: sh+). The interaction term is designated by *. Direction of differences between variables is indicated in brackets below F value. Significant values (p≤0.05) are indicated in bold. N = 100.

### Parasite-specific IgG levels increase with age in healthy people, but not in schistosome patients

**Figure 2:**
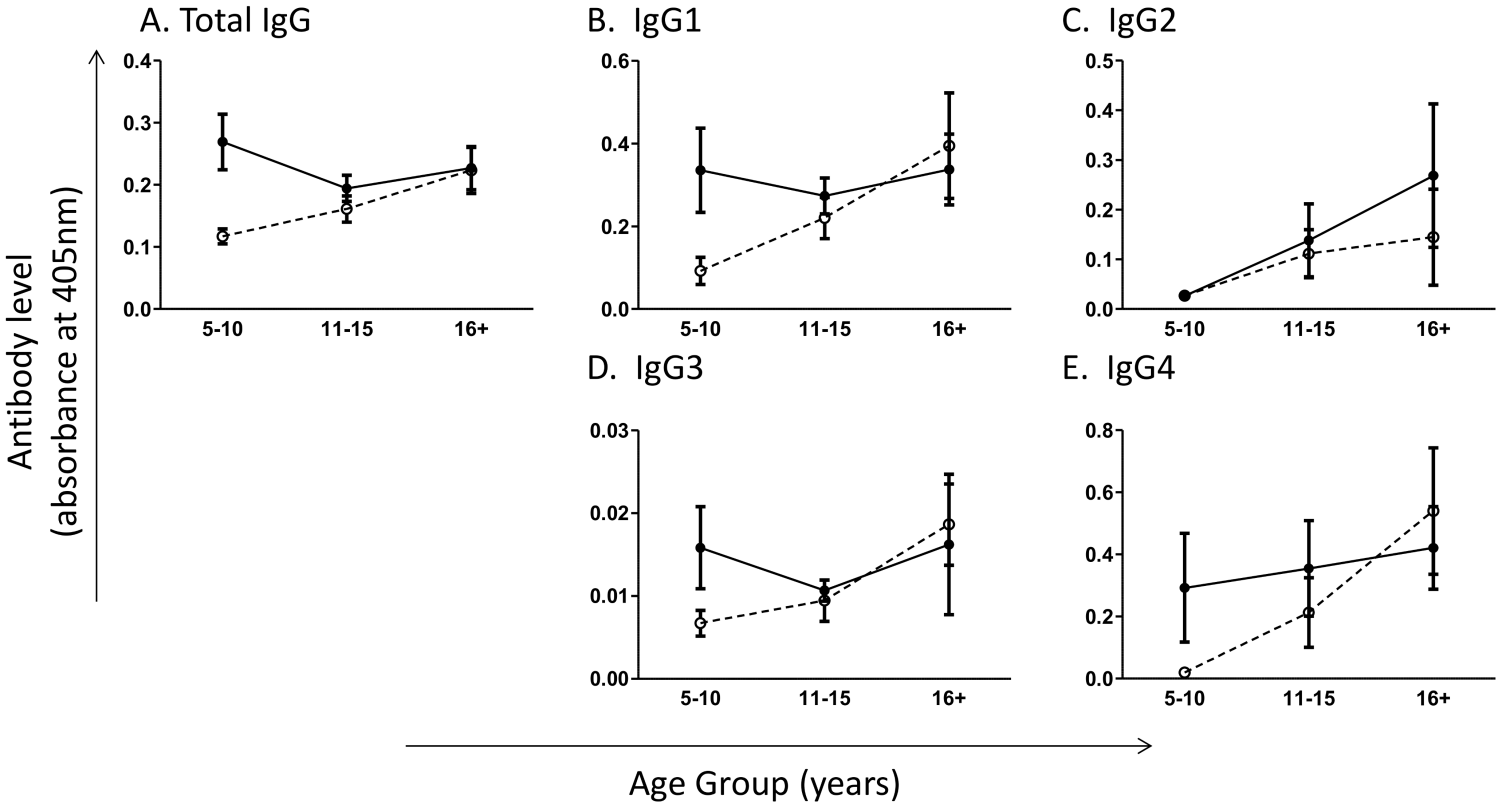
Total adult worm-specific IgG levels differ by age group in healthy participants but not schistosome patients. Adult worm specific (A) total IgG, (B) IgG1, (C) IgG2, (D) IgG3, (E) IgG4 responses by age group and infection status measured by ELISA. Open circles and dashed lines, healthy individuals; closed circles and solid lines, schistosome patients. Bars represent standard error of mean. Significant differences between schistosome infected and uninfected participants within age groups according to sub-group analysis are indicated by p: ** p≤0.005, *p≤0.05.

The relationship between SWAP-specific total IgG, as well as the IgG subclasses and infection status dependent on age was investigated. Total IgG, IgG1, IgG2 and IgG4, but not IgG3, significantly varied with host age, with total schistosome-specific IgG levels lowest in the age of peak schistosome infection, but the adult worm-specific subclasses IgG1, IgG2 and IgG4 highest in the oldest age group (Table 3). Overall, levels of parasite-specific IgG and IgG1 varied between healthy participants compared to schistosome infected people, with infected individuals having greater antibody levels compared to uninfected individuals (Table 3). However, only for total IgG, the effects of infection status varied with host age as indicated by the significant age group-infection status interaction term (Table 3). Figure 2 demonstrates the gradual increase in levels of schistosome-specific total IgG in healthy participants, while in schistosome infected patients, total IgG levels did not change significantly with age. For total IgG, IgG1, IgG3 and IgG4, but not IgG2, the youngest age group showed significant differences in antibody levels dependent on infection, with the infected individuals showing greater antibody levels compared to the uninfected individuals (Figure 2).

**Table 3:** Variables affecting adult worm specific IgG levels.

	*Dependent variable*				
*Independent variable*	*Total IgG*	*IgG1*	*IgG2*	*IgG3*	*IgG4*
Sex	2.695 (M>F)	0.687 (M>F)	0.377 (M<F)	0.006 (M<F)	1.063 (M>F)
Age group	**4.382 (1>2<3)**	**8.068 (1<2<3)**	**3.632 (1<2<3)**	2.699 (1<2<3)	**8.772 (1<2<3)**
Infection status	**9.040 (sh−<sh+)**	**5.302 (sh−<sh+)**	0.782 (sh−<sh+)	1.520 (sh−<sh+)	2.152 (sh−<sh+)
Age group* Infection status	**3.525**	2.718	0.256	2.200	0.564

F values from ANOVA for relationship between antibody and variables. Model accounts for variation in sex (male (M) or female (F)), age group (1∶5–10 years; 2: 11–15 years; 3: >16 years), and infection status (uninfected: sh−; infected: sh+). The interaction term is designated by *. Direction of differences between variables is indicated in brackets below F value. Significant F values (p≤0.05) are indicated in bold.

### CD16 expression level is related to parasite-specific IgG and IgG1 levels in healthy participants but not in schistosome patients

The relationship between intensity of CD16 expression on all monocytes and IgG levels against SWAP was investigated to test the hypothesis that innate cell (monocyte) phenotype is related to schistosome-specific acquired immune markers (IgG). As both CD16 expression and IgG antibody titres showed significant relationships with infection status, the population was partitioned by infection status prior to analysing the relationship between IgG and CD16 by regression analysis. This analysis showed that in healthy, uninfected individuals, expression levels of CD16 rose significantly with levels of total IgG and IgG1 after allowing for variation due to age and sex (Table 4). Expression levels of CD16 in infected individuals did not show a relationship with parasite-specific total IgG or IgG1. In addition, levels of IgG2, IgG3 and IgG4 did not show a relationship with CD16 expression levels in either infected or uninfected participants (Table 4).

**Table 4:** Relationship between SWAP specific IgG and CD16 expression according to infection status.

	CD16 MFI			
	Uninfected		Infected	
Antibody subclass	β (SE)	p-value	β (SE)	p-value
Total IgG	**0.346 (0.268)**	**0.007**	−0.188 (0.280)	0.280
IgG1	**0.282 (0.127)**	**0.032**	−0.069 (0.157)	0.704
IgG2	−0.001 (0.148)	0.993	0.231 (0.129)	0.196
IgG3	0.083 (0.661)	0.538	0.053 (0.610)	0.771
IgG4	0.101 (0.095)	0.448	−0.149 (0.091)	0.408

Regression (β) coefficients and the standard error (SE) and p-values from linear regression after accounting for sex and age differences. Significant p values and their associated β coefficients are indicated in bold.

## Discussion

Human schistosome-specific IgE and IgG1 responses have been shown to be associated with resistance to infection [41]–[43]. In order to understand naturally developed protective immune responses that can be targets for artificial induction through vaccination, previous studies in schistosomiasis have focussed on describing the interaction between IgE and cellular mediators of protective effector responses, such as eosinophils and macrophages. However, following Phase 1b vaccination trials on the human hookworm vaccine candidate, it was found that inducing an IgE response in naturally exposed people caused a pathological immune response, compromising the vaccine's safety [23]. Subsequently, there has been a shift from developing IgE-mediated helminth vaccines towards vaccines that induce the IgG1 and IgG3 subclasses. This study focused on determining the relationship between schistosome-specific IgG subclasses and IgG FcγRIIIa (CD16) on human monocytes, a cell type related developmentally to the macrophage and which has been shown to have a significant role in immune responses to experimental schistosome infection. Monocytes have previously been demonstrated to be involved in immune response to schistosome larvae [11], [12], [44], and as circulating cells interacting with adult schistosome stages in the venules, monocytes are a highly relevant, but under studied innate immune cell type in human schistosomiasis.

The relationship of increasing IgG subtypes with age, as well as the higher expression of IgG against adult worm antigen in infection, has previously been reported [20], [45]. Anti-SWAP IgG1 and IgG4 are both dominant antibody subclasses in human schistosomiasis, while IgG2 and IgG3 are detected at lower levels [46]. In particular, IgG1 is associated with protection or developing immunity [43] and IgG4 is associated with infection [47]. These schistosome specific responses observed here in both the older uninfected and infected groups confirm that both age groups have been exposed to schistosome infection. Although the age at which this exposure occurred in the uninfected group is not clear, the increasing levels of all the antibody subclasses with age represent the cumulative exposure to adult schistosome antigens throughout the population's lifetime. In human immuno-epidemiological studies, the uninfected, older individuals are classified as putatively resistant to infection via an immune-mediated mechanism [43], [48], and the gradual increase in parasite-specific total IgG and IgG1 in this group is consistent with schistosome-specific immune responses associated with protection [20], [22].

The presence of infection in the older individuals who are lifelong residents of this schistosome endemic area suggests that they are carrying chronic infections, a fact that has been corroborated by other studies conducted in the same area and including some of the same participants [49]. In particular, chitinase 3-like 1 protein, a marker of inflammation that has been linked to schistosome-related hepatic fibrosis [50], was found to be highest in the older people harbouring infection [49]. Indeed, with increasing age, and thus duration of exposure to schistosomes, differences in the immune system become more apparent, and studies investigating myeloid derived dendritic cells from members of the same residential area have, similar to results presented here, shown age related changes with infection [33]. In this study, members of the younger patient cohort will have had a shorter infection history compared to the older age groups, and therefore have experienced much less schistosomiasis associated pathology [5], [42], [51]. The differences in CD16 expression dependent on age and infection may therefore be indicating an altered immune activation status in relation to schistosome infection, and may indicate a potential link between CD16 expression and pathology. Indeed, the pattern of increasing CD16 expression with age, observed here in the healthy individuals, has previously been noted in other populations [38], [39], and CD16 expression has previously been reported to be upregulated with monocyte maturation and activation [15], [52].

The absence of a relationship between monocyte CD14 expression levels and schistosome infection is likely related to CD14 being a lipopolysaccharide (LPS) receptor, which is involved in immunity against bacterial challenge [53], [54], and therefore playing a less significant role in schistosome-specific responses induced by adult worm antigens.

In contrast to the significant relationship between CD16 and infection status shown here, we found no relationship between infection intensity and either CD14 or CD16 expression (data not shown), indicating that it was the presence of infection in the host that was important in this relationship rather than the burden of infection, a pattern that has been demonstrated in other immunological and pathological features of human schistosomiasis [49], [55].

The positive correlation observed between the protective IgG antibodies (total IgG and IgG1) and monocyte CD16 in uninfected individuals, indicates that the CD16 expression level on monocytes may be associated with protection against infection, in association with an activated monocyte phenotype. Observations from research into monocyte involvement in human HIV infection report on CD16 - IgG mediated ADCC activity [56], [57], and there may be a similar mechanism mediating protection in schistosomiasis, in particular involving CD16-IgG1 interactions. However, the precise role of any ADCC mechanism warrants further investigation. Importantly, consistent with results from mechanistic mouse experimental studies of schistosomiasis [19], this relationship suggests a link between the innate and adaptive arms of the immune system in the response to schistosomiasis, which may be important for furthering vaccine research efforts.

The lack of association between the other IgG subclasses, IgG2, IgG3, and IgG4 may be due to antibody properties such as length of memory response [58], [59] and lack of affinity between the Fcγ receptor and the antibody, as is the case with IgG4; or the relatively low levels of expression associated with schistosome infection, as is the case with IgG3; or a combination of these factors, as may be the case for IgG2.

Although the study cannot determine causality from the observations made here, transfection studies of a macrophage cell line demonstrated that chronic inflammation inhibits FcyRIIIa (CD16) glycosylation, in turn reducing the ability for CD16 receptor activation following IgG binding [60]. Thus, chronically infected individuals in this study may have deficiencies in their CD16 receptor contributing to continued infection. In addition to the Fcγ receptor, CD16, monocytes also express CD32 (also known as FcγRIIb) an inhibitory receptor [61], and CD64 (also known as FcγR1a), a high affinity receptor for IgG [62]. Differential expression of these receptors may further indicate the capability of monocytes to activate in response to infection, and may enlighten on the role of other IgG subtypes in schistosome infection.

Altogether, our study demonstrated that monocyte CD16 expression is associated with protection against schistosome infection. The level of CD16 in healthy individuals is positively associated with levels of total IgG and IgG1, antibodies which have previously been associated with resistance to infection. Conversely, in schistosome infected patients who are lifelong residents of a schistosome endemic area, CD16 expression is significantly reduced. This decrease in expression of a monocyte activation marker, combined with the lack of association with protective IgG, may be a result of an altered immune activation state in chronic schistosomiasis infection.
